# Ultra-high-frequency electrocardiography guided cardiac resynchronization therapy in a patient with an ambiguous electrocardiogram

**DOI:** 10.1016/j.hrcr.2026.01.023

**Published:** 2026-04-12

**Authors:** Uyên Châu Nguyên, Florien Klein, Leonard M. Rademakers

**Affiliations:** 1 Department of Cardiology, Maastricht University Medical Center+, Cardiovascular Research Institute Maastricht, Maastricht, The Netherlands; 2 Department of Cardiology, Catharina Ziekenhuis, Eindhoven, The Netherlands

**Keywords:** Cardiac resynchronization therapy, Conduction system pacing, Left bundle branch–optimized cardiac resynchronization therapy, Ultra-high-frequency electrocardiography, Noninvasive mapping

**Key Teaching Points**

• Ultra-high-frequency ECG (UHF-ECG) allows detailed assessment of ventricular activation in patients with ambiguous QRS morphology, providing insights beyond conventional surface ECG analysis.
• UHF-ECG enables real-time, within-patient comparison of different CRT modalities during implantation and optimization.
• Left bundle branch optimized cardiac resynchronization therapy has the potential to achieve superior electrical resynchronization compared with conventional biventricular pacing in selected patients.

## Introduction

Left bundle branch area pacing (LBBAP) and left bundle branch–optimized cardiac resynchronization therapy (LOT-CRT) have emerged as alternative approaches to conventional biventricular pacing (BiVP) for CRT. Observational data from the international LBBAP collaborative study group demonstrated that LOT-CRT achieved greater electrical resynchronization than BiVP.^1^ In the CSPOT trial, LOT-CRT provided hemodynamic improvement comparable with BiVP, particularly among patients with advanced conduction disease.^2^ Despite these favorable findings, electrophysiological differences between resynchronization modalities within the same patient remain unexplored. We present a case in which ultra-high-frequency electrocardiography (ECG) (UHF-ECG) enabled head-to-head comparison of ventricular activation during LBBAP, LOT-CRT, and conventional BiVP, allowing an individualized CRT strategy.

## Case Report

A 70-year-old man with symptomatic heart failure with reduced ejection fraction and inferolateral transmural scar owing to mixed ischemic and nonischemic cardiomyopathy was referred for CRT and implantation of an implantable cardioverter-defibrillator for primary prevention. The baseline ECG showed a QRS duration (QRSd) of 159 ms, QS complex in V1, broad and predominantly monophasic R waves in the lateral leads, and discordant T waves. This ambiguous morphology could be compatible with left bundle branch block according to the 2013 European Society of Cardiology criteria, although it does not clearly fulfill the 2021 European Society of Cardiology or Strauss definitions owing to the absence of distinct mid-QRS notching.

**Figure 1:**
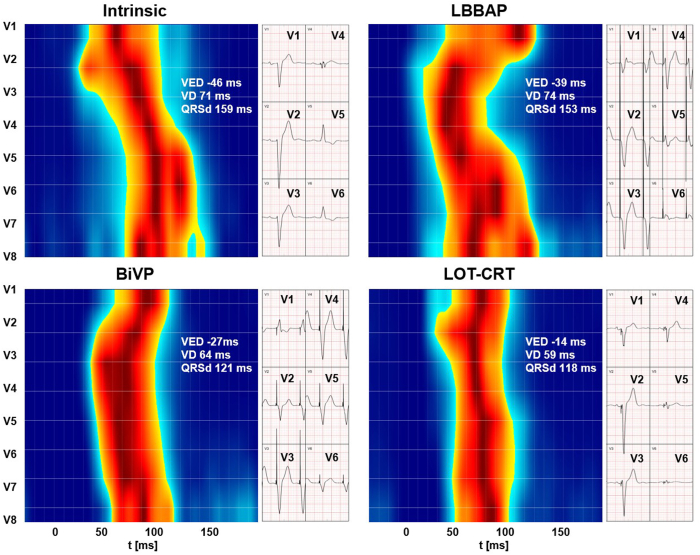
Ultra-high-frequency electrocardiography (UHF-ECG) depolarization map and conventional precordial ECG recorded during intrinsic activation, LBBAP, biventricular pacing, and LOT-CRT. Each horizontal row represents the normalized morphology of the UHF-QRS complex from 1 precordial lead (V1–V8), arranged along the vertical axis. Time is shown on the horizontal axis. Within each row, the maximum amplitude is depicted in *dark red* and the minimum amplitude in *dark blue*. VED is defined as the time interval between the earliest and the latest local activation times across all leads. VD represents the mean local activation duration across all leads. Elevated VD values indicate regions of slow conduction. BiVP = biventricular pacing; LBBAP = left bundle branch area pacing; LOT-CRT = left bundle branch–optimized cardiac resynchronization therapy; QRSd = QRS duration; VD = ventricular depolarization duration; VED = ventricular electrical delay.

UHF-ECG was performed during intrinsic activation and during several types of pacing, as illustrated in Figure 1, where time is depicted along the horizontal axis and the colored activation distribution across the precordial leads is displayed on the vertical axis. In UHF-ECG, leads V1 and V2 typically reflect right ventricular free wall and septal activation, whereas leads V6–V8 represent left ventricular free wall activation. The most common UHF-ECG parameters are mean ventricular depolarization duration (VD) and ventricular electrical delay (VED). VD reflects conduction velocity and is visually represented by the width of the activation distribution. It can be calculated per electrode or as the mean across all leads, with lower VD values corresponding to higher conduction velocity and higher VD values indicating a more nonhomogeneous substrate. The VD value shown in Figure 1 is averaged across all leads. VED is calculated as the maximal time difference between local activation times across all leads. UHF-ECG parameters are most often interpreted relative to a patient’s baseline, and no clear cutoff values are established, although reported values in healthy individuals range from 0 to 18 ms for VED and from 35 to 43 ms for VD.^3^

The upper left quadrant of Figure 1 shows that UHF-ECG during intrinsic activation demonstrated broad ventricular activation across all leads (VED, −46 ms; VD, 71 ms; QRSd, 159 ms), consistent with global conduction delay but without visual evidence of a distinct proximal conduction block, as reflected by a more gradual activation pattern from V1–V2 to V6–V8 rather than an abrupt transition. An LBBAP lead was implanted to assess whether electrical synchrony could be restored. After lead deployment, the ECG showed findings favorable for left bundle branch capture (R′ in V1, V6 R-wave peak time 95 ms, V6–V1 interpeak 60 ms), although no QRS morphology transitions were observed during programmed stimulation or threshold testing. However, the UHF-ECG, shown in the upper right quadrant, still demonstrated dyssynchronous ventricular activation (VED 39 ms, VD 74 ms, QRSd 153 ms). Therefore, we decided to implant an additional coronary sinus lead. Although BiVP (lower left quadrant) already substantially reduced VED, a more pronounced correction of electrical dyssynchrony was observed during LOT-CRT (lower right quadrant, VED −14 ms, VD 59 ms, QRSd 118 ms). Consequently, LOT-CRT was selected as the final resynchronization approach.

The patient was programmed with a ventricular-ventricular (VV) delay of 0 ms between the left bundle branch and coronary sinus leads. Only a limited number of studies have reported data on VV optimization in LOT-CRT. An abstract reporting CSPOT data,^1^ consisting predominantly of patients with intraventricular conduction delay with ischemic cardiomyopathy, a VV delay of 0 ms resulted in the most favorable electrical outcomes and the most favorable acute hemodynamic response.^4^ From a mechanistic perspective, VV optimization may depend on the underlying etiology and the type of capture achieved. Accordingly, computer simulations demonstrated that the electrically most optimal VV delay for LOT-CRT was 0 ms in simulations with severe intramyocardial conduction delay, whereas pre-excitation of the left bundle branch lead was optimal in simulations with mild left ventricular conduction delay.^5^

This image illustrates, using UHF-ECG, how different CRT modalities affect ventricular activation patterns. LOT-CRT achieved the most synchronous ventricular activation, surpassing that of BiVP and underscoring its potential to optimize CRT in patients with ambiguous baseline conduction abnormalities. To what extent this electrical optimization will translate into mechanical benefit and clinical improvement remains to be determined during follow-up.

## Disclosures

The authors have no conflicts of interest to disclose.
